# Prevention of Sexual Child Abuse: Preliminary Results From an Outpatient Therapy Program

**DOI:** 10.3389/fpsyt.2020.00088

**Published:** 2020-03-03

**Authors:** Tamara S. N. Wild, Isabel Müller, Peter Fromberger, Kirsten Jordan, Lenka Klein, Jürgen L. Müller

**Affiliations:** ^1^Clinic for Psychiatry and Psychotherapy – Forensic Psychiatry, Human Medical Center Göttingen, Georg-August-University Göttingen, Göttingen, Germany; ^2^Prevention of Sexual Abuse (PsM), Asklepios Psychiatric Clinic, Göttingen, Germany

**Keywords:** child sexual abuse, child sexual exploitation material, child pornography, sex offender, pedophilia, treatment, therapy, well-being

## Abstract

In Germany, access to outpatient treatment services devoted to the prevention of (further) sexual offenses against minors and child sexual exploitation material (CSEM) offenses is often limited. The therapy project “Prevention of Sexual Abuse” tries to fill this gap by providing treatment to patients with a self-reported sexual interest in children and adolescents, irrespective of whether or not they are pedophilic or prosecuted by the legal justice system. Within the project, a treatment manual was developed which specifically addresses dynamic risk-factors in child sexual abusers and CSEM offenders. The treatment manual was conceived to reduce recidivism risk and to contribute to the enhancement of the patients’ personal well-being. In this paper, results of the accompanying scientific research are presented: offense-supportive attitudes (*N* = 23), self-reported CSEM use (*N* = 10), emotional distress (*N* = 24), and participants’ subjective risk perception of committing (further) sexual offenses (*N* = 25) reduced during the course of treatment. A reduction of offense-supportive attitudes was further observed from pre-intervention to 1-year follow-up (*N* = 8). Changes with regard to self-efficacy, quality of life, participants’ self-perceived ability to control sexual impulses toward children and adolescents permanently, and several measures assessing different kinds of sexual recidivism did not, however, reach any level of significance. During an average observation period of 2.4 years, six patients confessed to have conducted new sexual exploitation material offenses, while no further sexual abuse cases were reported (*N* = 19). Due to the used research design and small sample sizes, treatment effects cannot be inferred and external validity is limited. This notwithstanding, results provide first evidence for a relationship between treatment participation and self-reported recidivism and psychological well-being.

## Introduction

In recent years, media attention has increasingly focused on child sexual abuse (CSA), hence raising the public awareness toward the need and importance of prevention programs for this specific offender group. In the literature, many definitions have been proposed for CSA (1, 2). What they have in common is that CSA does not need to include physical contact between a perpetrator and a child. Instead, the definitions refer to different kinds of sexual harassment that can occur on a continuum of power and control, ranging from non-contact sexual assault (e.g., exhibitionistic acts) to contact sexual assault (e.g., forcible rape). Moreover, the definitions also include internet sexual offending, that is, the exploitation of children online. As a consequence of the advent of new digital technologies and the growth of the Internet, possibilities to commit offenses against the sexual self-determination of minors from behind the computer screen have increased. Both the illicit distribution, acquisition, and possession of child sexual exploitation material (CSEM) and online grooming and solicitation, the initiation of online contacts with children with the intention of gratifying one’s sexual desire by means of the receipt of sexually explicit images or cybersex (3) fall in that category. Research indicates that the number of internet sexual offenses has increased (4, 5), aided by a phenomenon that can be traced to the ease of accessibility at affordable costs, while feeling secure due to the anonymity of the online environment [the so-called Triple A Engine: accessibility, affordability, and anonymity (6)].

Prevalence rates of sexual offending against minors are difficult to estimate. Data from both official arrest statistics and self-report studies may result in under-reporting. Different research groups have nevertheless attempted to examine the prevalence of CSA and CSEM offenses. For instance, Alanko et al. (7) were able to show that within a large sample of 3,909 Finnish men between the age of 21 and 43, 0.3% indicated to have had sexual contact with a person under the age of 16. In an online study with 8,718 participants (8), exclusive consumption of CSEM for sexual gratification was reported by 1.7% of subjects, exclusive CSA by 0.8%, and both CSA and CSEM offenses by 0.7%. Ten percent of the participants reported having any kind of online contact with minors, while 5.3% indicated they have had sexual online contact with minors (predominantly adolescents). The results further indicate that one third of sexual online contacts resulted in sexual offline meetings; however, also nonsexual online contacts sometimes resulted in sexual offline meetings (9).

A sexual interest in children is considered a risk factor for both the onset and progression of CSA (10). A sexual preference for children, usually of prepubertal or early pubertal age accompanied by persistent sexual fantasies and urges involving children over a period of at least 6 months, on which the individual has acted or which causes marked distress or interpersonal difficulty is described as a “pedophilia” according to ICD-10 (11). Nevertheless, a sexual interest in minors is neither a necessary nor a sufficient precondition for offenses against the sexual self-determination of children. In contrast, it is estimated that only 50% of child sexual abusers (CSAs) have a sexual orientation toward children (12). Accordingly, offenders should be provided with treatment irrespective of potential pedophilic interests.

Cognitive behavioral therapy programs applying the risk-need-responsivity principles and addressing dynamic risk factors have been shown to be most effective in the treatment of sex offenders (13, 14) and are of importance for a number of reasons: (1) having persistent sexual urges involving children can be experienced as markedly distressing and may therefore require treatment (15), (2) the committal of sexual offenses against children can entail a host of serious penalties, including substantial fines, probation, or jail sentences and can also result in the loss of significant others or social exclusion. (3) CSA is linked to a number of adverse consequences for the affected children. Indeed, minors who have been abused sexually may develop a variety of mental health problems such as affective disorders, suicidal behavior, alcohol, drug and medication dependence, social anxiety, conduct disorder, borderline personality disorder, posttraumatic stress disorder, eating disorders, especially bulimia nervosa, or an increased risk of revictimization (16–19). Moreover, there is evidence that children whose sexual abuse has been recorded and distributed on the internet additionally suffer once they realize that their indecent images cannot be removed from the Internet and that they are continuously being victimized by a large number of offenders (20, 21). In summary, the consequences of CSA and CSEM offenses are detrimental for both the offender and the victim, which emphasizes the importance of out-patient prevention programs.

### The Outpatient Treatment Facility “Prevention of Sexual Abuse” (PsM)

Originally, the provision of treatment for CSAs and child sexual exploitation material offenders (CSEMOs) in Germany was allocated to correctional institutions as well as mental health care services. However, while sex offenders who are sentenced to more than 2 years in prison receive mandatory treatment in correctional institutions, access to outpatient treatment services was often limited. The reasons for this are diverse and range from reservations regarding the patient group, fear of reputational damage, and a lack of willingness to cooperate with legal authorities [for an overview, see (22, 23); Brand, 2006, as cited in (22)]. By virtue of the limited access for the highly stigmatized offender group, in the last decade, a small yet growing number of specialized community programs targeting the prevention of (repeated) sexual assaults against minors were established throughout Germany (24, 25), one of them being the outpatient treatment facility “Prevention of Sexual Abuse”^1^ (PsM) in Göttingen (26). The PsM, which was established in 2011, addresses both men and women who are concerned about their sexual fantasies and behaviors toward children and adolescents, irrespective of whether they have already committed an offense against the sexual self- determination of children. In comparison to other specialized treatment centers, it is further irrelevant if clients fulfill the diagnostic criteria for pedophilia or are being prosecuted criminally. While voluntary participation, intrinsic motivation, willingness to change, and a high self-reported degree of psychological strain are mandatory inclusion criteria for (potential) CSAs and CSEMOs, offenders with probation conditions can nevertheless commence treatment. However, probation conditions cannot be met by participating in the program. The treatment program is funded by the State Government of Lower Saxony, the Human Medical Center Göttingen, and Asklepios Psychiatric Clinic Göttingen.

From July 2011 up to August 2019, 340 individuals have contacted the therapy project PsM. These callers included legal authorities, relatives, medical clinicians and psychotherapists, and others (e.g., legal guardians or priests). However, the majority of patients initiated contact with the PsM by themselves. In most cases, first contact was preceded by a house search and many of those concerned reported that they had been rejected by other specialized treatment programs because of this. In total, 122 patients started the diagnostic phase. From these patients, 83 have gone through the diagnostic phase, while seven patients still participate in it (current as of September 2019). Almost all patients were of male sex (*n* = 121), with a mean age of 37 years (*SD* = 11.9; range 18–77 years). Out of the 122 patients who were offered to start the diagnostic phase, 93 were involved in the legal justice system, 13 were undetected offenders, 14 dealt with sexual fantasies with minors, but had not yet committed a crime, and two suffered from pedophilia-themed obsessive–compulsive disorder. Interestingly, the proportion of fathers is higher among CSAs and mixed offenders compared to CSEMOs (39%, 50%, and 29%, respectively). This finding is consistent with previous reports (27) and emphasizes the importance of treatment programs in order to protect at-risk children from sexual exploitation.

A detailed description of the treatment program based on the first German treatment manual specifically addressing (potential) CSAs and CSEMOs (28) and first results can be found elsewhere (24, 26). In the following, we will present updated results from the ongoing accompanying scientific research as well as data on self-reported recidivism rates. In line with previous findings (24, 26) we expect improvements with regard to (1) general self-efficacy, (2) offense-supportive attitudes, (3) self-perceived overall emotional distress, (4) life satisfaction, (5) self-perceived ability to control their sexual impulses toward children and adolescents permanently, and (6) subjective risk perception of committing sexual offenses from pre-intervention to post-intervention and to 1-year follow-up. Additionally, we expect a reduction of the frequency of (7a) child and adolescent sexual abuse, and (7b) the consumption of child and adolescent sexual exploitation material use during the course of therapy.

## Method

### Participants

Participants were patients from our treatment facility who completed the whole treatment program and volunteered to take part in the study. The ethics committee of the Medical University Center Göttingen issued a positive vote and written informed consent was obtained from all participants. Depending on the therapy form, group size, and individual characteristics such as engagement in treatment (e.g., as indicated by homework compliance) or intellectual abilities, treatment length varied between several months and 2 years. Due to changes in data collection methodology, not all participants filled in every questionnaire and not all questionnaires were assessed at all four points in time [pre-intervention (baseline, T1), after the first half of the treatment manual had been completed (Ti; please note that measurements at this time point were not included in the analyses due to low case numbers), post-intervention (T2), and at 1-year follow-up (T3)]. As some participants just finished the treatment program recently or have dropped out after T2, little follow-up data is available. As a consequence, different analyses were conducted for participants with (a) pre-, post-, and follow-up intervention data and (b) pre- and post-intervention data. Accordingly, the two different samples will be described separately in the following two subsections.

#### Sample description for participants with pre-, post-, and follow-up intervention data (sample (a); *N* = 9)

Nine men aged 25 to 71 years, with a mean age of 44 years (*SD* = 13.9) and a mean IQ of 100 [range 80–123; *SD* = 14.8; (29, 30)] had filled in questionnaires at pre- and post-intervention and 1 year after treatment completion. Two had sexually abused a child, three had consumed CSEM, and five had committed both offense types. The majority of patients were involved in the justice system (*n* = 8), only one subject was an undetected offender. None of the patients were pedophilic according to ICD-10 criteria (missing values *n* = 1). However, the majority of patients (*n* = 5) fulfilled criteria for at least one psychiatric disorder. Three patients fulfilled criteria for affective disorders (F30-F39) and two patients were diagnosed with disorders of adult personality and behavior (F60-F69), one patient with mental and behavioral disorders due to psychoactive substance use (F10-F19), and another patient with neurotic, stress-related and somatoform disorders (F40-F49). Due to differences in personal backgrounds, participants either received individual or group therapy or both (*n* = 2, *n* = 5, and *n* = 2, respectively). Whenever necessary, additional sessions were offered to patients, meaning that treatment was not fully standardized.

#### Sample description for participants with pre-and post-intervention data (sample (b); *N* = 25)

The sample included 25 men aged 24 to 71 years, with a mean age of 41 years (*SD* = 11.8) and a mean IQ of 97 [range: 65-123; *SD* = 16.3; (29, 30)], who had sexually abused a child (*n* = 6), consumed CSEM (*n* = 12), committed both offense types (*n* = 6), or had not yet conducted any sexual offenses against children, but were afraid they might do so in the future (*n* = 1). The majority of patients were involved in the justice system (*n* = 22), a smaller proportion were undetected offenders (*n* = 2) or non-offenders (*n* = 1). The majority of patients (*n* = 22) were not pedophilic based on ICD-10 criteria. However, most fulfilled criteria for at least one psychiatric disorder. After consideration of various factors such as intellectual abilities, work schedule, and comorbid disorders, participants either received individual or group therapy or both (*n* = 5, *n* = 14, and *n* = 6, respectively).

**Tables 1** and **2** in the **Supplementary Material** provide an overview of demographic characteristics and psychiatric diagnoses of sample (a) and sample (b) grouped by offender status (involved in the justice system, undetected offenders, non-offenders) and offender type (CSAs, CSEMOs, individuals with both offenses), respectively.

## Materials

### Self-Efficacy

#### Aachen Self-Efficacy Questionnaire

The Aachen Self-Efficacy Questionnaire [ASF (31)] measures generalized self-efficacy as well as self-efficacy for achievement, social interactions and health-related behaviors. Twenty items (e.g., “I can trust my abilities”) have to be rated on a five-point Likert scale, ranging from 1 (“does not apply at all”) to 5 (“fully applies”). Total values can range between 20 and 100, with higher values reflecting greater subjective self-efficacy. Psychometric properties have been shown to be good. The internal consistency for the general scale is Cronbach’s α = .90, for the three subscales it is slightly lower (Cronbach’s α = .74-.84). Over a period of 8 weeks, test-retest reliability was *r*_tt_ = .66 (31).

### Offense-Supportive Attitudes

#### Bumby Molest Scale

Offense-supportive attitudes, that is, beliefs that excuse or justify sexual harassment, were measured with the Bumby Molest Scale [BMS (32); in the German version (33)]. The questionnaire consists of 38 items, an example being “Some sexual relationships with children are a lot like adult sexual relationships”. Items are rated on a four-point Likert scale, ranging from “strongly disagree” to “strongly agree”. The total value can vary between 38 and 152, with higher values representing stronger offense-supportive attitudes. In the original study, the Bumby Molest Scale showed good psychometric properties (Cronbach’s α = .97, *r*_tt_ = .84).

### Personal Well-Being

#### Symptom Checklist-90-Revised

To assess subjective symptoms and psychopathologic features, the Sympton Checklist-90-Revised [SCL-90_R (34); in the German version (35)], a self-report inventory comprised of 90 items on nine subscales (somatization, obsessive-compulsive, interpersonal sensitivity, depression, anxiety, hostility, phobic anxiety, paranoid ideation, and psychoticism) was used. All items have to be rated on a five-point Likert scale, ranging from 1 (“not at all”) to 4 (“extremely”). The Global Severity Index can be calculated to indicate overall emotional distress, with higher total values reflecting a greater subjective burden. Psychometric evaluations have reported adequate internal consistency (Cronbach’s α = .79 to .89), and acceptable to good test-retest reliability (35).

#### Life Satisfaction Questionnaire

The Life Satisfaction Questionnaire [FLZ (36)] measures general life satisfaction as well as satisfaction with health status, job, income, leisure time, partnership, relationship with one’s children, oneself, sexuality, friends and relatives, and housing. The ten different domains consist of seven items each, which results in 70 items that have to be rated on a seven-point Likert scale (1 = “very unsatisfied” to 7 = “very satisfied”). General life satisfaction is calculated as the sum of the seven subscales health status, income, leisure time, oneself, sexuality, friends and relatives, and housing and range between 49 and 343. Higher total values indicate a greater general life satisfaction, while higher subscale values reflect greater satisfaction in the specific domains. Psychometric properties were shown to be good. Validity has been demonstrated by factor analysis and internal consistency of the different subscales varies between Cronbach’s α = .82 and α = .95 (36).

### Subjective Sexual Self-Regulation

#### High Risk Situation Test

Subjective risk perception in a variety of situations (e.g., when alone with a child) was assessed by means of the High Risk Situation Test [HRST (37); German version (38)]. The questionnaire consists of 58 items that need to be rated on a five-point Likert scale, ranging from “low” to “extremely high”. The total value can vary between 58 and 290, with higher values representing an increased self-perceived risk to commit sexual offenses.

#### Coping Self-Efficacy Scale Related to Minors—Coping

The Coping subscale of the Self-Efficacy Scale Related to Minors [SESM-C (38)], consisting of 20 items, was used to measure the participants’ self-perceived ability to control their sexual impulses permanently. On a four-point Likert scale ranging from “not true” to “absolutely true”, participants have to indicate how certain they feel that they are able to control their sexual urges toward children or adolescents permanently in a variety of situations (e.g., when alone with a child). Lower scores represent greater deficits in the perceived ability to maintain self-control. Internal reliability was shown to be high (Cronbach’s α = .94).

### Self-Reported Sexual Offenses Against Children and Adolescents

In order to assess sexual offenses against minors, two self-report instruments were used—the *Sexual Behavior Involving Minors Scale* (SBIMS) (38) and the *Sexual Fantasies and Behaviors Questionnaire* (SPV) (26). The SBIMS was in use until February 2014 and was thereafter replaced by the SPV. Both instruments intend to measure the frequency of sexual contacts with minors and the consumption of sexual exploitation material depicting children and adolescents during the six months preceding the assessment. Furthermore, they both include items concerning the frequency of the occurrence of sexual fantasies including minors, which were not, however, used for the purpose of this paper.

#### Sexual Behavior Involving Minors Scale

The Sexual Behavior Involving Minors Scale [SBIMS (38)] is a questionnaire measuring the frequency of specified sexual behaviors during the last 6 months. To the best of our knowledge, normative data are not available. Two items concerning the frequency of sexual abuse of minors and the consumption of sexual exploitation material depicting minors were used for the purpose of this paper. Both items had to be rated on a five-point Likert scale ranging from “never” to “daily”. Higher scores represent more deviant sexual behavior.

#### Sexual Fantasies and Behaviors Questionnaire

Since the SBIMS does not differentiate between sexual offenses against children and those against adolescents, it was replaced by the Sexual Fantasies and Behaviors Questionnaire [SPV (26)]. The SPV is a self-developed unpublished inventory measuring the frequency of self-reported sexual fantasies of children and adolescents, sexual and non-sexual contacts with minors, and the consumption of child and youth sexual exploitation material during the six months prior to testing. For the purpose of this paper, four items were used [frequency of sexual abuse of (i) children, and (ii) adolescents, and frequency of sexual exploitation material use of (iii) children and (iv) adolescents]. All items were rated on a six-point Likert scale ranging from 1 (“never”) to 6 (“daily”), an example being “During the last 6 months, I have used child sexual exploitation material for sexual gratification”). Psychometric properties have not been assessed and normative data is not available.

### Individual Therapy Process

#### Questionnaire for General and Differential Single Therapy Sessions for Patients

To assess patients’ experiences with the therapeutic sessions, the Questionnaire for General and Differential Single Therapy Sessions for Patients [STEPP (39)] was administered. The instrument includes three subscales (motivational clarification, problem activation, and therapeutic relationship), and comprises of 12 items. Statements such as “What I learned today will help me deal with my difficulties in the future” have to be rated on a seven-point Likert-scale, ranging from 1 (“not true at all”) to 7 (“absolutely true”). Higher scores on the subscales reflect greater subjectively experienced progresses in the different domains. Internal consistencies have been shown to be good (r_tt_ = .76 to r_tt_ = .89) (39).

### Analyses

All analyses were computed using the software SPSS version 26.0 (40). To compare mean differences between pre- and post-intervention (T1 and T2) and between pre-, and post-intervention, and 1-year follow-up (T1, T2, and T3), Wilcoxon signed-rank tests for matched pairs as well as Friedman repeated measures tests were performed.

## Results

### Self-Efficacy

Self-Efficacy was assessed using the Aachen Self-efficacy Questionnaire (31). Sample (a): Our expectation that participants’ self-reported general self-efficacy would increase over time was not confirmed. In a sample of six participants, changes from pre-intervention to post-intervention and 1-year follow-up (*M*_pre_ = 3.5; *SD*_pre_ = 0.4; *M*_post_ = 3.7; *SD*_post_ = 0.6; *M*_follow-up_ = 3.6; *SD*_follow-up_ = 0.6) were not significant (χ^2^(2) = 1.000, *p* = .607). Sample (b): Pre- and post-intervention data were further compared in a sample of 19 participants (*M*_pre_ = 3.6; *SD*_pre_ = 0.4; *M*_post_ = 3.8; *SD*_post_ = 0.5). Also here, participants’ self-reported general self-efficacy did not change significantly (*Z* = −1.289, *p* = .197).

### Offense-Supportive Attitudes

**Figure 1:**
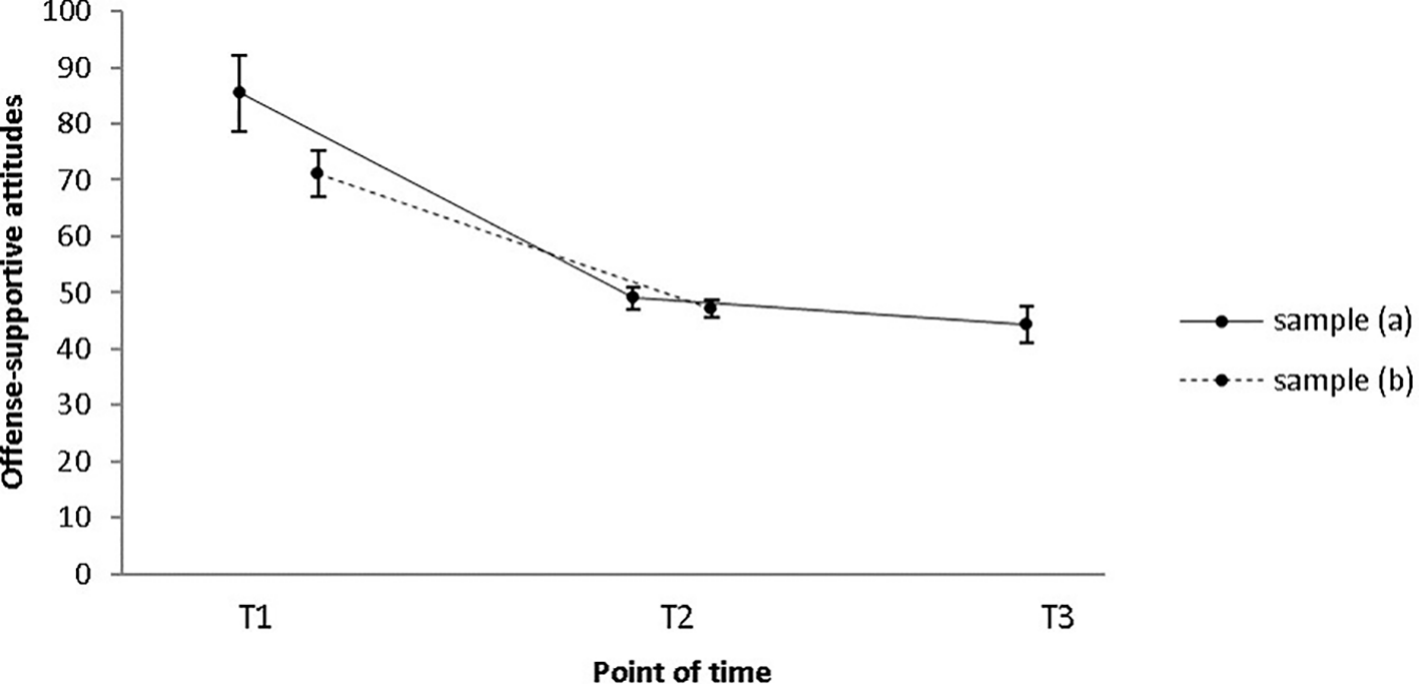
Mean offense-supportive attitude scores as measured by the BMS significantly decreased from T1 to T2 and from T1 to T3 in sample (a) and from T1 to T2 in sample (b). Error bars indicate standard errors. T1 = pre-intervention; T2 = post-intervention; T3 = 1-year follow-up.

Changes with regard to offense-supportive attitudes were assessed using the Bumby Molest Scale (32). Sample (a): On a descriptive level, a reduction of offense-supportive attitudes was observed from pre-intervention to post-intervention to 1-year follow-up (*M*_pre_ = 85.4, *SD*_pre_ = 19.2; *M*_post_ = 49.1, *SD*_post_ = 5.6; *M*_follow-up_ = 44.3, *SD*_follow-up_ = 9.3; see **Figure 1**). Results of a Friedman test with *N* = 8 show that this reduction is significant (χ^2^(2) = 13.067, *p* = .001). Bonferroni-corrected post-hoc-tests indicated a significant reduction from pre- to post-measurement (*p* = .018; *r* = .49) and from pre- to follow-up-measurement (p = .003; *r* = .57) but not from post- to follow-up-measurement (*p* = 1.0). Sample (b): The reduction of self-reported offense-supported attitudes from pre- to post-intervention remained significant in a larger sample of *N* = 23 with a large effect size (*M*_pre_ = 71.0; *SD*_pre_ = 19.6; *M*_post_ = 47.1; *SD*_post_ = 7.5; *Z* = −3.817, *p* < .001; *r* = .56; see **Figure 1**). Median offense-supportive attitude score was 69 at pre-treatment and 48 at post-treatment.

### Personal Well-Being

**Figure 2:**
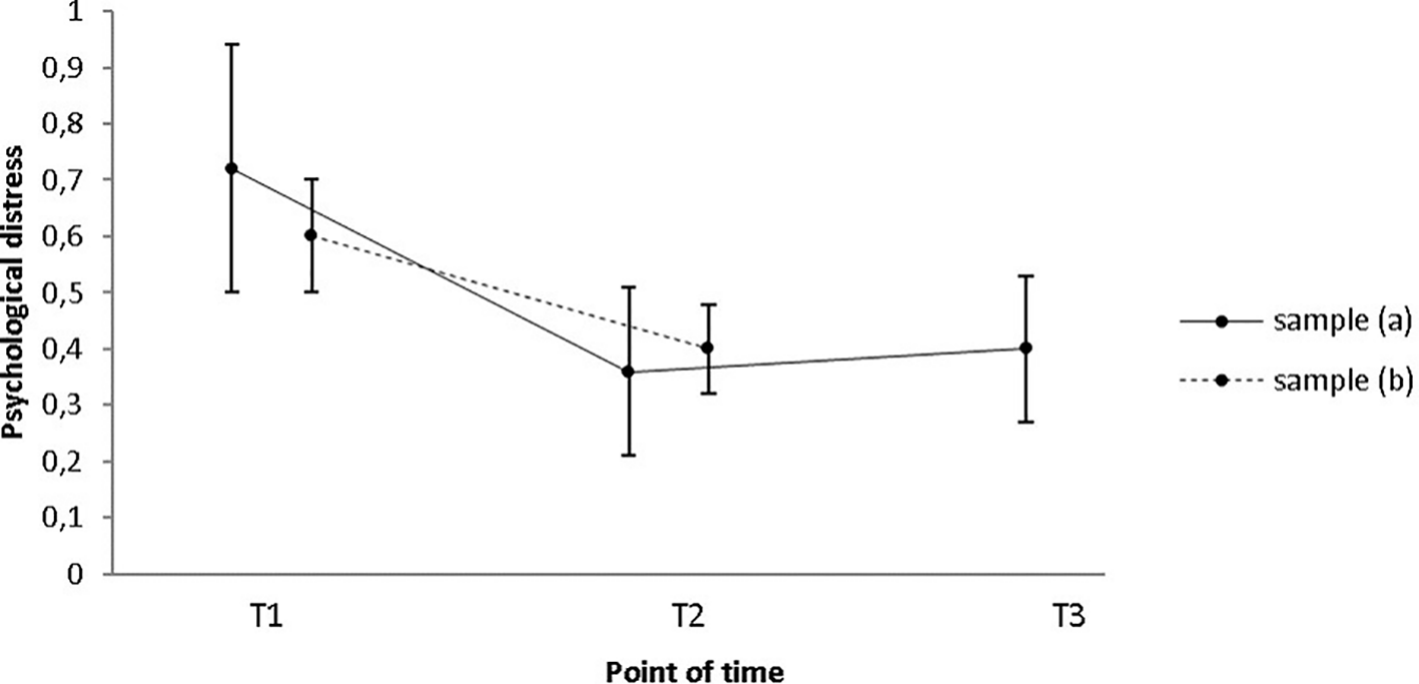
Participants’ mean self-perceived psychological distress score as measured by the SCL-90-R only decreased significantly from T1 to T2 in sample (b), while no change occurred in sample (a). Error bars indicate standard errors. T1 = pre-intervention; T2 = post-intervention; T3 = 1-year follow-up.

The Symptom Checklist-90-Revised (34) was used in order to assess participants’ self-perceived overall psychological distress and results will be provided in the following. Sample (a): Changes with regard to participants’ self-perceived overall psychological distress were not significant between the three times of measurement (*N* = 9; *M*_pre_ = 0.72; *SD*_pre_ = 0.65; *M*_post_ = 0.36; *SD*_post_ = 0.44; *M*_follow-up_ = 0.41; *SD*_follow-up_ = 0.39; χ^2^(2) = 3.765. *p* = .053), but a trend was evident (see **Figure 2**). Sample (b): Results of a pre/post-comparison with *N* = 24 further indicate a statistically significant change with a medium effect size (*M*_pre_ = 0.59; *SD*_pre_ = 0.49; *M*_post_ = 0.4; *SD*_post_ = 0.39; *Z* = −2.159, *p* = .031; *r* = 0.31; see **Figure 2**). Median emotional distress score decreased from 0.4 at pre-intervention to 0.3 at post-intervention. The reduction remained significant when participants receiving psychotherapy for comorbid disorders were excluded from the analysis (*N* = 19; *M*_pre_ = 0.5; *SD*_pre_ = 0.39; *M*_post_ = 0.28; *SD*_post_ = 0.29; *Z* = −2.675, *p* = .007; *r* = 0.35).

The Life Satisfaction Questionnaire (36) was employed to measure participants’ self-reported life satisfaction. Sample (a): Participants’ self-reported life satisfaction did not increase significantly from pre-treatment to post-treatment to 1-year follow-up in a small sample of five participants (*M*_pre_ = 201.0; *SD*_pre_ = 31.2; *M*_post_ = 229.0; *SD*_post_ = 45.0; *M*_follow-up_ = 238.4; *SD*_follow-up_ = 39.9; χ^2^(2) = 4.778, *p* = .092). Sample (b): Also in a sample of 20 participants, no significant improvement regarding participants’ self-reported life satisfaction from pre- to post-intervention was evident (*M*_pre_ = 241.5; *SD*_pre_ = 45.6; *M*_post_ = 254.9; *SD*_post_ = 38.3; *Z* = −1.456, *p* = .145).

### Subjective Sexual Self-Regulation

**Figure 3:**
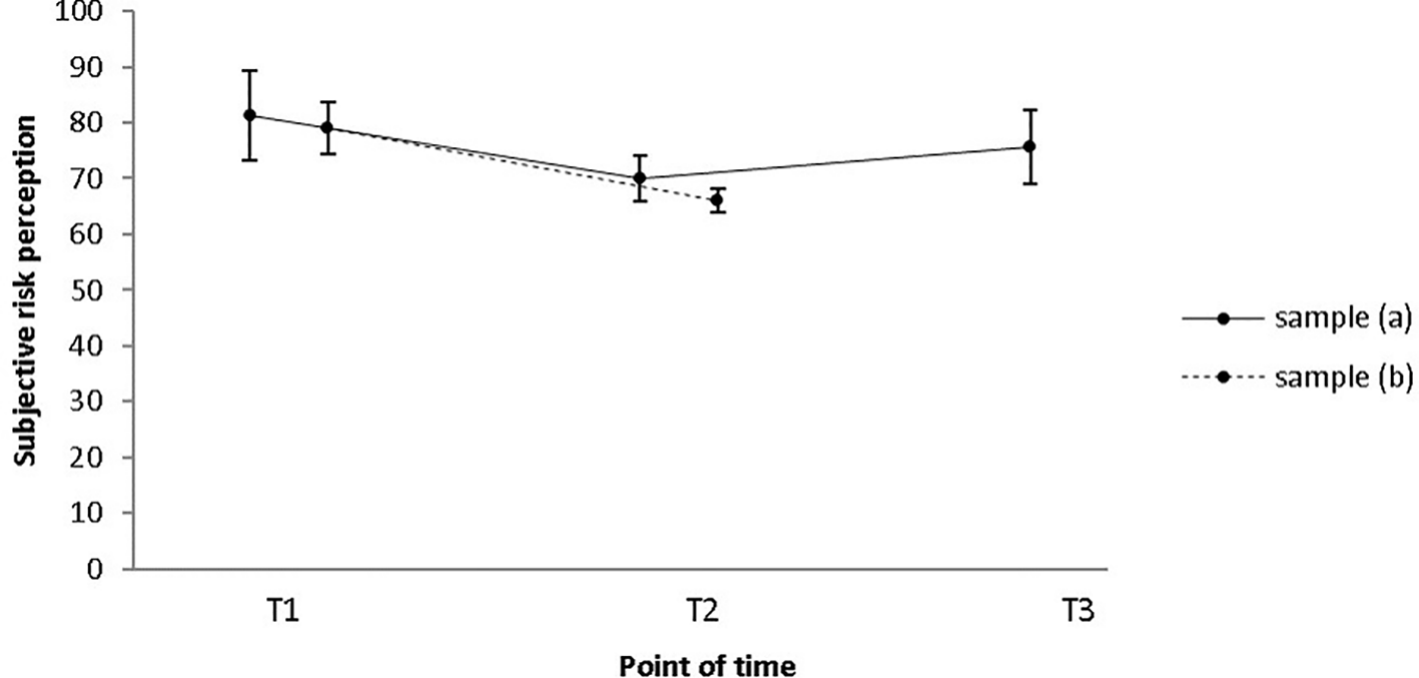
Participants’ mean subjective risk perception score as measured by the High Risk Situation Test (HRST) only decreased from T1 to T2 in sample (B), while no change occurred in sample (a). Error bars indicate standard errors. T1 = pre-intervention; T2 = post-intervention; T3 = 1-year follow-up.

Participants’ subjective risk perception was assessed by means of the High Risk Situation Test (37). Sample (a): Concerning participants’ subjective risk perception, pre-, post-, and 1-year follow-up data were available for a small sample of *N* = 9. However, contrary to our expectations, results do not indicate a statistically significant difference between the three times of measurement (*M*_pre_ = 81.3; *SD*_pre_ = 24.4; *M*_post_ = 70.0; *SD*_post_ = 12.0; *M*_follow-up_ = 75.6; *SD*_follow-up_ = 20.0; χ^2^(2) = 2.242, *p* = .326; see **Figure 3**). Sample (b): In a sample of *N* = 25, a decrease of the participants’ subjective risk perception of committing sexual offenses can be observed. Results further demonstrate that this decrease was significant with a medium effect size (*M*_pre_ = 79.0; *SD*_pre_ = 22.9; *M*_post_ = 66.1; *SD*_post_ = 10.8; *Z* = −2.937, *p* = .003; *r* = .42; see **Figure 3**). Median risk perception score decreased from 68 at pre-intervention to 61 at post-intervention.

Changes regarding participants’ self-perceived ability to control deviant sexual impulses were measured using the Coping Self-Efficacy Scale Related to Minors – Coping (38). Sample (a): The increase of the participants’ self-perceived ability to control deviant sexual impulses permanently from pre- to post-intervention and 1-year follow-up was not significant in a sample of nine participants (*M*_pre_ = 61.2; *SD*_pre_ = 12.7; *M*_post_ = 69.2; *SD*_post_ = 10.1; *M*_follow-up_ = 67.8; *SD*_follow-up_ = 13.4; χ^2^(2) = 3.765, *p* = .152). Sample (b): Descriptively, an increase was observed in a sample of *N* = 23 (*M*_pre_ = 65.4; *SD*_pre_ = 11.6; *M*_post_ = 71.3; *SD*_post_ = 9.1). This increase was, however, not statistically significant (*Z* = −1.845, *p* = .065). Nevertheless, there was a trend in the expected direction with an increase of the median score from 68 at pre-treatment to 75 at post-treatment.

### Self-Reported Sexual Offenses Against Children and Adolescents

**Figure 4:**
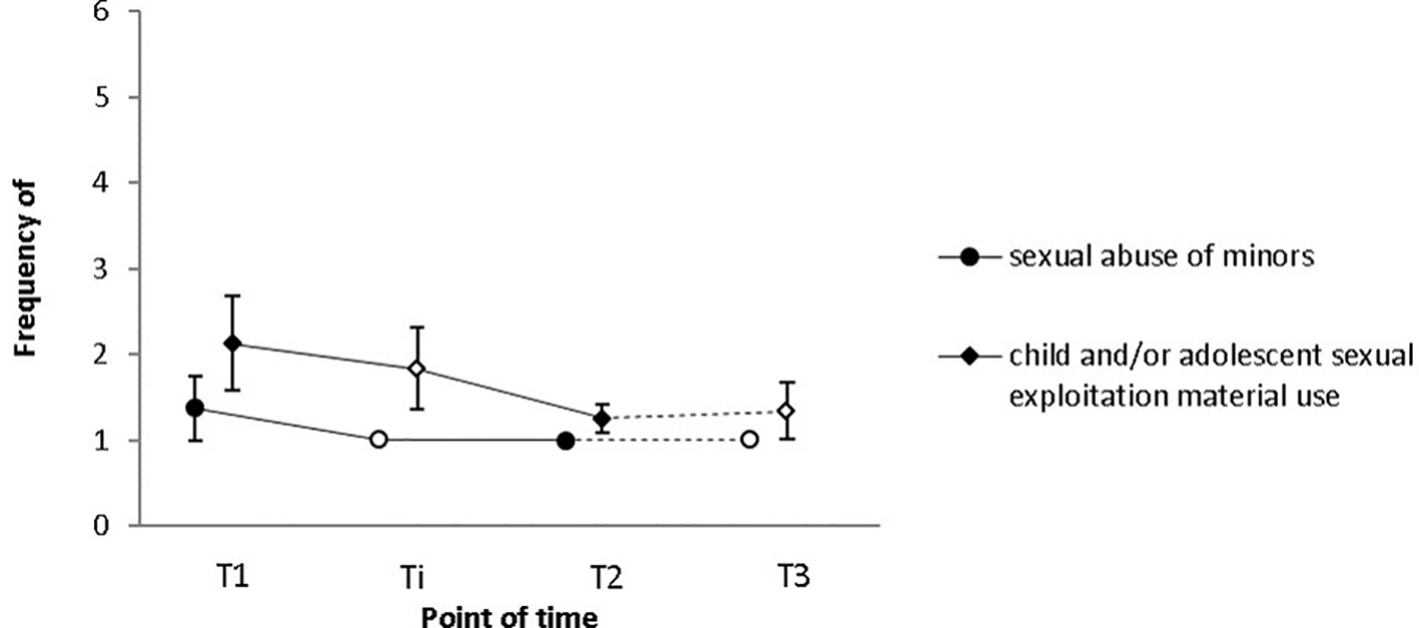
Neither the frequency of child/adolescent sexual abuse, nor the frequency of child/adolescent sexual exploitation material use changed significantly from T1 to T2 as indicated by Sexual Behavior Involving Minors Scale (SBIMS) scores. Frequency was indicated on a five-point Likert Scale (1 = never; 2 = few times a month; 3 = monthly; 4 = weekly; 5 = daily). Empty squares depict data points excluded from the analyses due to low case numbers (Ti: *N* = 6, T3: *N* = 3). Error bars indicate standard errors. T1 = pre-intervention, Ti = intervention, T2 = post-intervention, T3 = 1-year follow-up.

Results concerning deviant sexual behavior as measured by the Sexual Behavior Involving Minors Scale [SBIMS (38)] are described below. Sample (a): As follow-up data was only available for three participants, we refrained from conducting analyses with these data. Sample (b): Before the SPV (26) had been put into use, the SBIMS was administered to eight participants to assess the frequency of (1) self-reported child or adolescent sexual abuse, and (2) child or adolescent sexual exploitation material use during the 6 months prior to the assessment. Only few participants had filled in the SBIMS halfway through the intervention (*N* = 6). As a consequence, this measurement point was excluded from the analyses. Notwithstanding this, **Figure 4** depicts data from all four measurement points. Changes over time were insignificant with regard to both the frequency of sexual abuse offenses (*M*_pre_ = 1.4; *SD*_pre_ =1.1; *M*_post_ = 1; *SD*_post_ = 0; *Z* = −1, *p* = .317), and child and/or adolescent sexual exploitation material use (*M*_pre_ = 2.1; *SD*_pre_ = 1.6; *M*_post_ = 1.3; *SD*_post_ = 0.5; *Z* = −1.289, *p* = .197).

**Figure 5:**
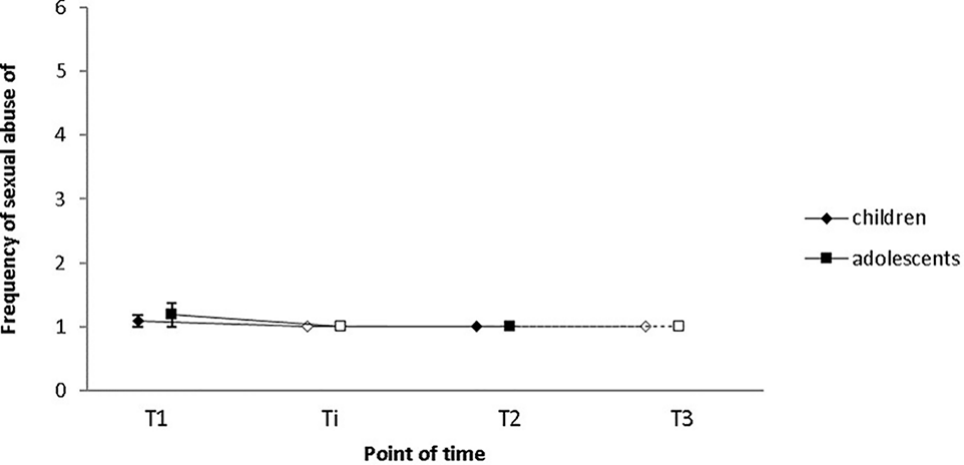
Self-reported child and adolescent sexual abuse did not decrease significantly from T1 to T2 as indicated by Sexual Fantasies and Behaviors Questionnaire (SPV) scores. Frequency was indicated on a six-point Likert Scale (1 = never; 2 = less than once a month; 3 = one to three times a month; 4 = once a week; 5 = multiple times a week, 6 = daily). Empty squares indicate data points excluded from the analysis due to low case numbers (Ti: N = 9, T3: N = 3). Error bars indicate standard errors. T1 = pre-intervention, Ti = intervention, T2 = post-intervention, T3 = 1-year follow-up.

Results regarding deviant sexual behavior as measured by the Sexual Fantasies and Behaviors Questionnaire [SPV (26)] are provided in the following. Sample (a): As follow-up data was only available for three participants, we refrained from conducting analyses with these data. Sample (b): The SPV was administered to assess the frequency of (i) self-reported child and adolescent sexual abuse, and (ii) child and adolescent sexual exploitation material use during the 6 months prior to testing. None of the participants filling in the questionnaire had also filled in the SBIMS, meaning that in total, data on self-reported recidivism was provided by 19 subjects. Only data from participants who filled in the questionnaire at both pre- and post-intervention were considered for this paper. As only part of these participants filled in the questionnaire halfway through the intervention (*N* = 9), this measurement point was again excluded from the analyses. However, to give a better picture of relapses with regard to offline and online offenses during treatment and on the long term, **Figures 4** and **5** depict data from all four measurement points (T1 = pre-intervention; Ti = intervention; T2 = post-intervention; T3 = 1-year follow-up).

**Figure 6:**
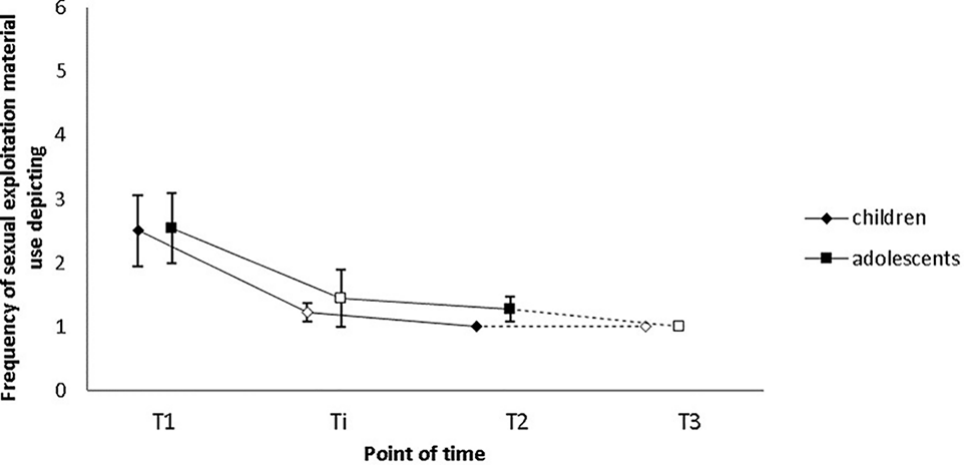
Participants’ frequency of self-reported child sexual exploitation material consumption decreased significantly from T1 to T2 as indicated by SPV scores. Changes in the frequency of self-reported adolescent sexual exploitation material consumption were not significant, but a trend was evident. Frequency was indicated on a six-point Likert Scale (1 = never; 2 = less than once a month; 3 = one to three times a month; 4 = once a week; 5 = multiple times a week, 6 = daily). Empty squares depict data points excluded from the analysis due to low case numbers (Ti: *N* = 9, T3: *N* = 3). Error bars indicate standard errors. T1 = pre-intervention, Ti = intervention, T2 = post-intervention, T3 = 1-year follow-up.

(i) The SPV was administered to assess the frequency of self-reported CSA during the 6 months prior to testing. In total, data of 11 subjects were assessed before and after the intervention, of three of these subjects 1-year follow-up data are also available. Nine participants had consumed child or youth sexual exploitation material and the remaining two participants had conducted sexual offenses against children (less than once a month; *n* = 1) and adolescents (one to three times a month; *n* = 1). After the first half of treatment, at post-intervention and at 1-year follow-up, none of the participants reported any child or adolescent sexual offenses in the prior 6 months, suggesting that both CSAs and CSEMOs did not conduct any (further) child sexual offenses. The change from T1 to T2 did neither reach the level of significance with regard to CSA (*M*_pre_ = 1.1; *SD*_pre_ = 0.3; *M*_post_ = 1.0; *SD*_post_ = 0; *Z* = −1.000, *p* = .317; see **Figure 5**) nor with regard to adolescent sexual abuse (*M*_pre_ = 1.2; *SD*_pre_ = 0.6; *M*_post_ = 1.0; *SD*_post_ = 0; *Z* = −1.000, *p* = .317; see **Figure 5**). This result, however, does not come as a surprise given that two participants had conducted contact offenses during the 6 months prior to T1 (CSAr: *n* = 1; adolescent sexual abuser: *n* = 1).

(ii) Ten participants, two CSAs and eight CSEMOs had reported their frequency of CSEM use during the 6 months prior to the beginning of the intervention and after the intervention. During the 6 months before T1, six participants had consumed such materials, three less than once a month, and another three multiple times a week. No (further) offenses were reported at post-intervention or at the 1-year follow-up. Results of a Wilcoxon signed-rank test indicate that the reduction from T1 to T2 is significant with a large effect size (*M*_pre_ = 2.5; *SD*_pre_ = 1.8; *M*_post_ = 1.0; *SD*_post_ = 0; *Z* = −2.251, *p* = .024; *r* = .50; see **Figure 6**). Median consumption score decreased from 2 (less than once a month) at pre-intervention to 1 (never) at post-intervention. Eleven participants, two CSAs and nine CSEMOs had additionally reported their frequency of youth sexual exploitation material use at T1 and T2. At pre-intervention, six subjects had consumed such images and videos: one less than once a month, two one to three times a month, one once a week, one multiple times a week, and another one daily. At post-intervention, only two participants had relapsed, one of them less than once a month and the other one to three times a month. This decrease was not significant as calculated by a Wilcoxon signed-rank test (*M*_pre_ = 2.5; *SD*_pre_ = 1.8; *M*_post_ = 1.3; *SD*_post_ = 0.6; *Z* = −1.876, *p* = .061), but a trend in the expected direction could be observed (see **Figure 6**). At 1-year follow-up, none of the three participants had conducted (further) offenses.

In total, three child or adolescent sexual abusers, 12 sexual exploitation material offenders, and four men who had conducted both types of offenses provided data on the frequency of recidivism. **Table 1** gives an overview on recidivism rates regarding sexual abuse and sexual exploitation material use based on SPV (26) and SBIMS (38) scores. For the purpose of this overview, item 1 and 2 of the SPV (CSA and adolescent sexual abuse) as well as item 3 and 4 (usage of sexual exploitation material depicting children and usage of sexual exploitation material depicting adolescents) were merged (sexual abuse of minors and usage of sexual exploitation material depicting minors). Both during treatment and at 1-year follow-up, none of the 19 participants reported to have abused a child or adolescent. However, six participants (31.58%) had consumed sexual exploitation material during treatment and one participant (16.67%) during the 1-year follow-up period. One should keep in mind, however, that the two questionnaires may have measured slightly different constructs due to differences in the wording of the items (e.g. “comsumption of sexual exploitation material depicting sexual activities with minors” (SBIMS) versus “consumption of sexual exploitation material depicting children for sexual gratification” (SPV). Additionally, the questionnaires did not cover the whole treatment duration. Accordingly, the relapse rates presented in **Table 1** may underestimate real recidivism rates.

**Table 1:** Participants’ recidivism rates during treatment participation and at one-year follow-up.

	Number of patients sexually abusing children or adolescents		Number of patients consuming sexual exploitation material		Number of patients conducting both offense types	
	during treatment (Ti-T2)	during one-year f-up (T3)	during treatment (Ti-T2)	during one-year f-up(T3)	during treatment (Ti-T2)	during one-year f-up (T3)
Child and/or adolescent sexual abusers	0/3	-/-	0/3	-/-	0/3	-/-
Child and/or adolescent sexual exploitation material users	0/12	0/4	5/12	1/4	0/12	0/4
Individuals with both offense types	0/4	0/2	1/4	0/2	0/4	0/2
**All participants**	**0/19**	**0/6**	**6/19**	**1/6**	**0/19**	**0/6**

Recidivism rates during an average observation period of 2.4 years were calculated using SPV and SBIMS scores (26, 40). Recidivism during treatment is based on Ti and T2 scores, while recidivism during the one-year follow-up period is based on T3 scores. Please note that the questionnaires did not cover the whole observation period and that all information is based on self-reports and could not be compared with criminal records. f-up = follow-up.

### Drop-Outs

A substantial number of participants dropped out during treatment participation. In total, 59 out of 122 patients discontinued study participation prematurely, 13 during the diagnostic phase, 3 before treatment start, 31 during treatment, and 2 after the end of treatment but before T2. Besides, 10 participants were expelled from treatment. **Table 3** in the **Supplementary Material** provides an overview of numbers and reasons for drop-outs and expulsions during different phases of the therapeutic process. Reasons for drop-outs and expulsions were determined based on participants’ self-reports or in cases where patients dropped out without providing any reason, the therapists’ subjective perception. Whenever more than one reason was applicable, the reason considered most important was recorded.

## Discussion

The aim of this study was to examine the relationship between participation in our treatment program for (potential) CSAs and CSEMOs and a variety of psychological variables. By means of self-report measures, we assessed changes from pre- to post-intervention and 1-year follow-up. Results indicate that offense-supportive attitudes, emotional distress, the use of CSEM, and participants’ subjective risk perception of committing (further) sexual offenses decreased significantly from pre- to post-intervention, and in the case of offense supportive attitudes also from pre-intervention to 1-year follow-up. The remaining measures of quality of life, self-efficacy, participants’ self-perceived ability to control sexual impulses toward children and adolescents permanently, and the frequency of child and adolescent sexual abuse, and adolescent sexual exploitation material use did not reach a level of statistical significance, although in some instances, results indicate trends in the expected direction. In the following, the main results will be discussed, and alternative explanations will be offered. In addition, suggestions for future research directions will be made.

### Self-Efficacy

To our knowledge, self-efficacy has yet to be identified as a dynamic risk factor for sexual recidivism. Nevertheless, self-efficacy has been shown to be associated with continued abstinence among drug users (41, 42) and smokers (43) and has additionally been related to reduced dropout rates from treatment (42). Therefore, we tried to enhance self-efficacy to increase treatment adherence. Contrary to our expectations, general self-efficacy did not increase significantly during the course of the intervention. However, a closer look at the data reveals that none of the participants had a below average sense of self-efficacy in the beginning of treatment. Instead, 63% of patients scored within the normal range and 37% scored above average [based on percentile ranks provided by (31)]. Accordingly, there was not much room for improvement.

Even though former participants did believe in their ability to succeed the face of adversity, individuals with a low sense of self-efficacy may possibly participate in the program in the future. In that case, it would be interesting to assess if treatment participation is associated with an increase of self-efficacy and if changes with regard to that construct are related to abstinence and drop-out rates.

### Offense-Supportive Attitudes

Offense-supportive attitudes are an empirically supported risk factor for sexual recidivism (10) and are therefore considered as an important treatment target. Examples for offense supportive attitudes include, but are not limited to, victim-blaming, misperceiving social cues as sexual, or failure to take responsibility for one’s actions. A whole treatment module in our treatment program is dedicated to the change of offense-supportive attitudes, and throughout the intervention, cognitive restructuring is continuously being applied. Based on Quayle et al.’s recommendations for the therapeutic work with Internet sex offenders (44), further emphasis is placed on cognitive distortions that are most evident in CSEMOs. In sample (a), offense-supportive attitudes decreased significantly from pre-intervention to post-intervention and 1-year follow-up. From post-intervention to 1-year follow-up, a decrease could only be observed on a descriptive level. However, this could be due to the fact that the sample size for the pre/post/follow-up comparison was rather small or because the improvement that was achieved during therapy remained stable over time.

Also in the larger sample (b) with 23 participants, offense-supportive attitudes decreased significantly from pre- to post-intervention [*M*_pre_ = 71.0 (*SD* = *19.6), M*_post_ = 47.1 (*SD* = 7.5)]. This is in line with the results of another out-patient prevention program (45), in which the score decreased significantly from 70.88 (*SD* = 17.11) to 63.30 (*SD* = 16.68). However, even though the control group’s cognitive distortions in this study remained stable over time, as indicated by a Wilcoxon signed-rank test, the time×group interaction did not reach a level of significance (46). Accordingly, due to the absence of a waiting-list control group, it cannot precluded that in our study, reductions also result from time effects.

While at pre-intervention, our patients descriptively scored higher than a sample of incarcerated non-familial child molesters, nonsexual offenders, and non-offending controls in the community (*M* = 71.0 versus *M* = 66.0, *M* = 52.3, and *M* = 51.8, respectively) (47), at post-intervention, they scored lower than any of the other groups. The finding that our patients had lower scores than non-offending controls in the community is especially surprising as offense-supportive attitudes have repeatedly been associated with recidivism risk in sexual offenders in general, and also for child molesters in particular (10, 48). Accordingly, the question arises of whether the decrease of offense-supportive attitudes as measured by the BMS really reflects changes in attitudes, or if the difference from pre- to post-intervention is, at least partially, caused by impression management or a growing understanding of the theoretical construct. In future research, it may therefore be useful to include measures of social desirability such as the Social Sexual Desirability Scale of the Multiphasic Sex Inventory (49) to control for this possible effect.

### Personal Well-Being

Our expectation that patients’ overall psychological distress decreases from pre- to post-measurement was supported. This does not come as a surprise since most of our patients are known to the justice system. In the majority of cases, participants started the program right after they had been confronted with a house search or an invitation to police interrogation. In the case of undetected offenders, strong feelings of distress may have driven them to seek help. According to self-reports, patients felt distressed for a variety of reasons, including feelings of guilt and shame, fear of being left by their partners, losing their children and housing, or imprisonment, and the fear that their offense is made public and they may be excluded socially. Additionally, many thought they were the only individuals dealing with these kinds of problems, which was perceived as onerous.

During therapy, we encouraged participants to develop methods to deal with their deviant sexual fantasies, desires, and urges. Step by step, we tried to assist them in learning how to satisfy their needs in a legal and prosocial way and in elaborating strategies allowing them to better handle social situations, and negative emotions such as anxiety, feelings of depression, et cetera. Of course, there are alternative explanations for the decrease of subjective burden. For instance, as can be seen in the sample description (see **Supplementary Material**: **Tables 1** and **2**), a substantial amount of patients suffered from at least one psychiatric disorder in the beginning of treatment. In many cases, we recommended to undergo a second therapy to target the(se) disorder(s). A small amount of patients indeed sought and received help from another mental health care professional (*n* = 5). As this may have positively affected overall psychological distress, we repeated the analysis without these patients. The results, however, remained stable. Additionally, as we did not include a control group, we cannot preclude that the observed changes were caused by time effects (50). This assumption would, however, at the very least be plausible, given that participants may have adjusted to their new living conditions 2 years following the start of treatment and—in many cases—2 years after (initial) contact with law enforcement authorities.

Compared to psychological distress, life satisfaction did not improve significantly during the course of the intervention. However, a closer look at the data revealed that pre-intervention life satisfaction score was normal or above average for the majority of patients [75%; based on general population norms from (36)]. Further research with a larger data set will have to reveal if the PsM’s therapeutic concept has the potential to increase life satisfaction for those who score below-average.

### Subjective Sexual Self-Regulation

During therapy, patients identified their individual risk situations and worked on their self-control strategies for such situations. Based on earlier results (24, 26), we predicted that participants’ subjective risk perception of committing sexual offenses would decrease during the course of treatment. Closer examination of the data reveals that risk perception at pre-intervention already was relatively low (as indicated by a score approaching the minimum score). Nevertheless, a significant decrease was observed from pre- to post-intervention, but not to 1-year follow-up. Interpreting this result is difficult: the question arises of whether subjective risk perception decreased because participants were more comfortable with their impulse control skills or because they underestimated the risk posed by certain situations after the end of treatment. Regardless of the limited informative value of the questionnaire, we nevertheless believe that it is a useful tool to determine risky situations in the beginning of treatment. This information may then be used during the therapeutic process to develop appropriate risk prevention strategies.

As compared to subjective risk perception, participants’ self-perceived ability to control sexual impulses toward children and adolescents permanently did not increase significantly during the course of therapy, a finding which is in contrast to the results obtained by another out-patient prevention program (45). Notwithstanding this, a trend in the expected direction was evident, and there was also relatively little room for improvement, since participants’ self-perceived self-control abilities had already been rather high in the beginning of the intervention [sample (a): *M*_pre_ = 61.2, sample (b): *M*_pre_ = 65.4; highest possible score: 80]. This being said, further analyses with more data will have to be conducted in order to assess if subjective risk perception does indeed decrease during the course of treatment, while self-control abilities remain stable. If this was the case, treatment may have devastating consequences, as patients may underestimate the risk posed by certain situations without being able to deal better with such situations. Despite this, results should be interpreted with caution as both questionnaires that were used [SESM-C (51), HRST (37)] are self-report measures and therefore reflect views of the patients rather than a structured risk assessment tool applied by mental health care professionals.

### Self-Reported Sexual Offenses Against Children and Adolescents

Baseline rates of sexual recidivism in CSAs are estimated to lie between 13.7% and 17.5% (14, 52), while recidivism rates in a sample of 541 registered CSEMOs with an average follow-up time of 4.1 years for new contact offenses and CSEM offenses added up to 4% and 7%, respectively (53). At least for CSAs, it was demonstrated that relapses occur less often in treated sex offenders (13, 14, 52). While in large-scale meta-analyses, sexual recidivism occurred in 9.5% to 10.1% of cases (14, 52), in another outpatient prevention program, 20% of CSAs relapsed based on self-reports (45). In our sample, none of the seven CSAs or mixed-offenders reported to have committed further child sexual offenses during treatment or at 1-year follow-up (average observation period: 2.4 years; range: 1.04 – 4.5; *SD* = 0.85). Additionally, none of the twelve exclusive CSEMOs committed any first-time offenses. Levenson and Prescott (54) criticize the focus on absolute measures of recidivism (i.e., relapse vs. no relapse) and suggest to include relative measures, such as changes in the frequency of relapses in treatment evaluations. However, contrary to our expectations, the frequency of self-reported child and adolescent sexual abuse did not decrease from pre- to post-intervention. This does not, however, mean that our treatment concept does not have the desired effect. During the six months prior to treatment start, only three participants had self-reportedly committed sexual offenses against minors [SBIMS (38): *n* = 1; SPV (26): *n* = 2]. More data needs to be collected to draw better conclusions on whether or not the treatment concept is associated with self-reported recidivism. Especially long-term data would be of special relevance to examine if potential treatment effects are stable over time. This being said, one should keep in mind that all information gained is based on self-reports and could not be compared with criminal records. As impression management or social desirability may have affected the results, measures such as the Social Sexual Desirability Scale of the Multiphasic Sex Inventory (49) or the Impression Management Subscale of the Balanced Inventory of Desirable Responding (55) should be included in the test battery to control for this possible effect.

There is an ongoing dispute on the effectiveness of child sexual offender therapy for CSEMOs. The core Sex Offender Treatment Programme (SOTP) has been found to increase recidivism in Internet sex offenders (56) and the question arose whether general sex offender treatments should be adapted to the needs of this specific offender group. Indeed, CSAs and CSEMOs differ with regard to a number of dynamic risk factors, that is, factors that have been shown to correlate with recidivism risk and that should be addressed by therapeutic interventions [for an overview, see (10)]. For instance, in a study in which pedophilia was assessed by means of penile plethysmography, Seto, Cantor, and Blanchard (57) could demonstrate that as compared to CSAs, a higher proportion of CSEMOs is pedophilic (35% versus 61%, respectively). In a systematic review, it could further be outlined that pedophilic interest is even more pronounced in mixed offenders (58). Moreover, CSEMOs demonstrate less offense-supportive attitudes, emotional congruence with children, and antisocial features as indicated by a smaller number of prior offenses and less problems with supervision (27). Elliot et al. (59) further found that CSEMOs score lower on cognitive impulsivity, a component of impulsivity characterized by quick cognitive decision-making. As a consequence, specialized treatment protocols specifically addressing relevant dynamic risk factors for CSEMOs may need to be developed (60). Based on previous results from our research group (24, 26), we nevertheless expected that participants’ frequency of self-reported child and adolescent sexual exploitation material consumption would decrease over the course of the intervention. However, only the frequency of child sexual exploitation material use as determined by the SPV (26) decreased significantly from pre- to post-intervention, while the mean frequency of sexual exploitation material use depicting adolescents (SPV) or minors in generel [SBIMS (38)] remained stable. In total, 19 subjects provided information on CSEM-related relapses. During treatment, six participants relapsed at least once, among them five exclusive CSEMOs and one mixed offender. Additionally, 1 year after the end of treatment, one exclusive CSEM offender who had already relapsed during treatment reported to have had a relapse (16.67%). In other words, during the average observation period of 2.4 years, 31.58% of subjects re-offended at least once. In another outpatient prevention program, 91% of individuals with a history of CSEM offenses committed further online offenses during the one year treatment period (45). Interestingly, the self-reported recidivism rates reported in our paper and in the paper of this other prevention program are substantially higher than the recidivism rates in studies, in which recidivism was measured using criminal records. This finding is in line with the literature suggesting that both institutionalized sex offenders and non-incarcerated paraphiliacs disclose an enormous amount of undetected sexual aggression in self-report studies (61, 62). Interestingly, the number of confessed sex crimes and nonsex crimes in such studies even seems to exceed the number of registered offenses (63). We therefore hypothesize that CSEM-related recidivism in the literature may often be underestimated as a result of the assessment method used. Notwithstanding this, we cannot preclude that treatment may have increased recidivism in online offenders as in the case of the core SOTP (56). Moreover, patients may have disclosed a small proportion of their lapses only since they may have been afraid that their therapists break confidentiality in the case of frequent relapses.

Initially, the PsM was designed as a 1-year program with three times of measurement (T1: pre-intervention, Ti: intervention, T2: post-intervention). Accordingly, questionnaires assessing relapses during the 6 months prior to assessment were used to cover the whole treatment duration. However, due to strong interindividual differences regarding general cognitive ability, motivational factors, and dynamic risk factors, treatment length had to be adapted. Additionally, a follow-up measurement one year after the end of treatment was included. Consequently, the results reported in this paper do not cover the whole observation period. To avoid such gaps in the future, we will administer the SPV half-yearly. Furthermore, we started to include a second relative measure of recidivism (changes in the intensity of sexual offenses), since the combination of the two measures frequency and intensity offer a means to evaluate improvement in outcome more precisely (54).

## Limitations

The empirical results reported in this paper should be considered in the light of some limitations and must therefore be interpreted with caution. For instance, due to inclusion criteria imposed by the authors, the results reported in this paper cannot be generalized to all CSAs and CSEMOs in forensic psychiatry. Furthermore, due to the low sample size, even external validity with regard to (potential) out-patient offenders who participate voluntary, are intrinsically motivated, willing to change, and have a high self-reported degree of psychological strain is limited. Because of changes in the data collection methodology, not all participants filled in every questionnaire and not all questionnaires were assessed at all four points in time. The follow-up data is especially sparse, meaning that at the moment, it is not possible yet to predict long-term changes. Additionally, dropouts may have resulted in attrition bias. Due to the long treatment length of our program (approximately 2 years for the majority of patients), some of the participants dropped out for personal reasons (e.g., move to another city, incarceration or need to be transferred to an inpatient therapy setting). Moreover, some other participants dropped out for reasons that we cannot ascertain. Dropouts occurred in all phases of the treatment program, including the diagnostic phase, the intervention, or in-between the end of the intervention and the 1-year follow-up data collection. Given that the majority of patients dropped out due to motivational reasons, it may be necessary to put a stronger emphasis on behavioral techniques enhancing motivation during the diagnostic phase and in the beginning of the intervention.

Relapse rates may have been underestimated since the two questionnaires used to assess recidivism did not cover the whole treatment period. What is more, they may have measured slightly different constructs due to differences in the wording of the items. Furthermore, social desirability in the form of over-reporting desirable or under-reporting undesirable cognitions, feelings, or behavior may have affected the results of the study. This tendency may have additionally been reinforced as the same therapists performed both the treatment and the accompanying scientific research. Unfortunately, since we do not have access to criminal records, we cannot compare the information given by the patients. Moreover, one could argue that participants are not completely open as they may fear that the information confessed is passed to the police. However, a substantial amount of patients voluntarily brought their indictments and some even confessed past offenses which are, according to their own testimony, unknown to the police and the prosecution authorities. This suggests that participants believe in confidentiality and trust in our treatment team and is in line with the finding that participants judge the therapeutic relationship to be good and supportive [as measured by the Questionnaire for General and Differential Single Therapy Sessions for Patients (39)].

Another limitation of the study was that we did not include a waiting-list group to control for effects of time. As a consequence, we cannot preclude that significant changes over time are attributed to random factors such as spontaneous remission or regression to the mean rather than the described treatment program (50). However, due to the patients’ self-reported high psychological strain, the potential detrimental consequences of not receiving treatment, and long waiting times of approximately 2 years, we have decided it would be unethical not to offer treatment to everybody in need. Changes over time may have also been affected by contemporaneous treatments of comorbid disorders. As can be seen in the sample description, a substantial amount of patients suffered from comorbid disorders, especially affective and/or personality disorders. As the PsM therapy concept is not disorder-specific, but does instead address dynamic risk factors related to sexual recidivism, comorbid patients are often recommended to undergo an additional disorder-specific psychotherapy. Some of them were also getting treated medically, e.g. with SSRIs in case of an existent affective disorder. Although this concerned only a small number of patients, comorbid disorders as well as medical and psychological treatment may have had confounding effects on our results. Even though accompanying psychiatric treatment did not seem to be a confounding variable with regard to overall emotional distress, we suggest to address this question more deeply in future research with larger sample sizes.

## Conclusion

In this paper, we examined the relationship between the participation in our treatment program for CSAs and CSEMOs and a variety of psychological variables. Over time, offense-supportive attitudes, self-reported child sexual exploitation material use, emotional distress, and participants’ subjective risk perception of committing (further) sexual offenses reduced significantly. During an average observation period of 2.4 years, six out of 19 online offenders relapsed, while no further offline offenses were reported. Although the results provide first evidence for a relationship between treatment participation, self-reported recidivism and psychological well-being, results remain preliminary and must be interpreted with caution. Sample sizes were small, no waiting-list control group was included and participants were a subgroup of sex offenders with specific characteristics. Further research with a larger sample and a different research design will be necessary before firm conclusions can be drawn.

## Data Availability Statement

The datasets generated for this study will not be made publicly available. Our data are highly sensitive (deviant sexual interest/child sexual offenses) and cannot be anonymized.

## Ethics Statement

This study was reviewed by the ethics committee of the University Medical Center Göttingen and a positive vote was issued. Written informed consent was obtained from all participants.

## Author Contributions

IM, TW, and JM conceived the topic of the paper. TW and IM conducted literature searches, performed statistical analyses and wrote the first draft of the manuscript. PF, KJ, LK, and JM critically revised the manuscript and approved the final version.

## Funding

The authors would like to thank the Lower Saxony State Office for Social Affairs, Youth and Family, the Human Medical Center Göttingen, and the Asklepios Psychiatric Clinic Göttingen for their financial support enabling this work. We further acknowledge support by the Open Access Publication Funds of Göttingen University and are grateful for our patients who consented that their data is being used for research purposes.

## Conflict of Interest

The authors declare that the research was conducted in the absence of any commercial or financial relationships that could be construed as a potential conflict of interest.

## Footnotes

1 https://www.asklepios.com/goettingen/experten/schwerpunkte/psm/
