# Supplementary material for: Prevention of Sexual Child Abuse: Preliminary Results From an Outpatient Therapy Program
Source: Front Psychiatry. 2020 Mar 3;11:88. doi: 10.3389/fpsyt.2020.00088 (PMC7063028; doi:10.3389/fpsyt.2020.00088)
Supplement: Supplementary file 1 [file Table_1.docx]

Table 1

*Demographic characteristics and psychiatric diagnoses of participants grouped by offender status*

|  | Involved in the justice system | | Undetected offenders | | No offense committed | |
| --- | --- | --- | --- | --- | --- | --- |
|  | Sample (a)  *n* = 8 | Sample (b)  *n* = 22 | Sample (a)  *n* = 1 | Sample (b)  *n* = 2 | Sample (a)  *n* = 0 | Sample (b)  *n* = 1 |
| Age mean score (*SD*) | 46 (12.8)  (range 31-71) | 43 (11.3)  (range 28-71) | 25 | 25 (0.7)  (range 24-25) | - | 37 |
| IQ mean score (*SD*) (1,2) | 98 (13.0)  (range 80-123) | 95 (15.7)  (range 65-126) | 123 | 117 (8.5)  (range 111-123) | - | 109 |
| Being in a relationship | 38% | 41% | 0% | 50% | - | 0% |
| F65.4: Pedophilia | 0% | 14% | 0% | 0% | - | 0% |
| No ICD-10 diagnosis | 38% | 36% | 0% | 0% | - | 0% |
| One ICD-10 diagnosis | 38% | 27% | 0% | 50% | - | 100% |
| More than one ICD-10 diagnosis | 13% | 36% | 100% | 50% | - | 0% |
| ICD-10: F00-F09 | 0% | 0% | 0% | 0% | - | 0% |
| ICD-10: F10-F19 | 13% | 9% | 0% | 0% | - | 0% |
| ICD-10: F20-F29 | 0% | 0% | 0% | 0% | - | 0% |
| ICD-10: F30-F39 | 25% | 23% | 100% | 100% | - | 100% |
| ICD-10: F40-F49 | 13% | 18% | 0% | 0% | - | 0% |
| ICD-10: F50-F59 | 0% | 0% | 0% | 0% | - | 0% |
| ICD-10: F60-F69 | 13% | 68% | 100% | 50% | - | 0% |
| ICD-10: F70-F79 | 0% | 9% | 0% | 0% | - | 0% |
| ICD-10: F80-F89 | 0% | 0% | 0% | 0% | - | 0% |
| ICD-10: F90-F98 | 0% | 0% | 0% | 0% | - | 0% |
| ICD-10: F99 | 0% | 0% | 0% | 0% | - | 0% |

*Note*. The three groups “Involved in the justice system”, “Undetected offenders” and “No offense committed” were created based on patients’ self-reports. Due to small case numbers, differences in offender groups are only presented descriptively and no formal statistical comparisons were undertaken. Clinical diagnoses were based on ICD-10 and were clustered into the following categories: Organic, including symptomatic, mental disorders (F00-F09), Mental and behavioral disorders due to psychoactive substance use (F10-F19), Schizophrenia, schizotypal and delusional disorders (F20-F29), Affective disorders (F30-F39), Neurotic, stress-related and somatoform disorders (F40-F49), Behavioral syndromes associated with physiological disturbances and physical factors (F50-F59), Disorders of adult personality and behavior (F60-F69), Mental retardation (F70-F79), Disorders of psychological development (F80-F89), Behavioral and emotional disorders with onset usually occurring in childhood and adolescence (F90-F98), Unspecified mental disorder (F99). As percentage scores are rounded to the nearest whole percentage point, “No ICD-10 diagnose”, “One ICD-10 diagnose”, and “More than one ICD-10 diagnose” do not sum up to 100% for individuals involved in the justice system.
