# Supplementary material for: Prevention of Sexual Child Abuse: Preliminary Results From an Outpatient Therapy Program
Source: Front Psychiatry. 2020 Mar 3;11:88. doi: 10.3389/fpsyt.2020.00088 (PMC7063028; doi:10.3389/fpsyt.2020.00088)
Supplement: Supplementary file 2 [file Table_2.docx]

Table 2

*Demographic characteristics and psychiatric diagnoses of participants grouped by offense type*

|  | Child sexual abusers | | Child sexual exploitation material offenders | | Individuals with both offense types | |
| --- | --- | --- | --- | --- | --- | --- |
|  | Sample (a)  *n* = 2 | Sample (b)  *n* = 6 | Sample (a)  *n* = 3 | Sample (b)  *n* = 12 | Sample (a)  *n* = 5 | Sample (b)  *n* = 6 |
| Age mean score (*SD*) | 44 (12.7)  (range 35-53) | 39 (8.9)  (range 31-56) | 37.7 (7.6)  (range 31-46) | 38 (11.0)  (range 24-62) | 49 (16.6)  (range 25-71) | 49 (14.8)  (range 25-71) |
| IQ mean score (*SD*) (1,2) | 106 (24.7)  (range 88-123) | 91 (21.9)  (range 65-123) | 87 (8.1)  (range 80-96) | 94 (14.5)  (range 66-111) | 104 (12.7)  (range 88-123) | 105 (12.2)  (range 88-123) |
| Being in a relationship | 50% | 50% | 33% | 34% | 40% | 50% |
| F65.4: Pedophilia | 0% | 33% | 0% | 8% | 0% | 0% |
| No ICD-10 diagnosis | 50% | 33% | 33% | 33% | 20% | 33% |
| One ICD-10 diagnosis | 50% | 33% | 33% | 25% | 40% | 33% |
| More than one ICD-10 diagnosis | 0% | 33% | 33% | 42% | 20% | 33% |
| ICD-10: F00-F09 | 0% | 0% | 0% | 0% | 0% | 0% |
| ICD-10: F10-F19 | 0% | 17% | 0% | 0% | 20% | 17% |
| ICD-10: F20-F29 | 0% | 0% | 0% | 0% | 0% | 0% |
| ICD-10: F30-F39 | 0% | 0% | 67% | 50% | 20% | 17% |
| ICD-10: F40-F49 | 50% | 17% | 0% | 17% | 20% | 17% |
| ICD-10: F50-F59 | 0% | 0% | 0% | 0% | 0% | 0% |
| ICD-10: F60-F69 | 0% | 33% | 33% | 33% | 20% | 17% |
| ICD-10: F70-F79 | 0% | 17% | 0% | 8% | 0% | 0% |
| ICD-10: F80-F89 | 0% | 0% | 0% | 0% | 0% | 0% |
| ICD-10: F90-F98 | 0% | 0% | 0% | 0% | 0% | 0% |
| ICD-10: F99 | 0% | 0% | 0% | 0% | 0% | 0% |

*Note.* The three groups “Child sexual abusers”, “Child sexual exploitation material offenders”, and “Individuals with both offense types” were created based on patients’ self-reports. Due to small case numbers, differences in offender groups are only presented descriptively and no formal statistical comparisons were undertaken. Clinical diagnoses were based on ICD-10 and were clustered into the following categories: Organic, including symptomatic, mental disorders (F00-F09), Mental and behavioral disorders due to psychoactive substance use (F10-F19), Schizophrenia, schizotypal and delusional disorders (F20-F29), Affective disorders (F30-F39), Neurotic, stress-related and somatoform disorders (F40-F49), Behavioral syndromes associated with physiological disturbances and physical factors (F50-F59), Disorders of adult personality and behavior (F60-F69), Mental retardation (F70-F79), Disorders of psychological development (F80-F89), Behavioral and emotional disorders with onset usually occurring in childhood and adolescence (F90-F98), Unspecified mental disorder (F99). As percentage scores are rounded to the nearest whole percentage point, “No ICD-10 diagnose”, “One ICD-10 diagnose”, and “More than one ICD-10 diagnose” do not sum up to 100% for child sexual abusers and individuals with both offense types.

1. Petermann F ed. *WAIS-IV. Wechsler Adult Intelligence Scale – Fourth Edition.* (2012).

2. Aster M, Neubauer A, Horn R. Wechsler Intelligenztest für Erwachsene WIE. Deutschsprachige Bearbeitung und Adaption des WAIS-III von David Wechsler. *Frankfurt/Main, Ger Harcourt Test Serv* (2006) doi:http://dx.doi.org/10.1038/srep18573
