# Supplementary material for: Prevention of Sexual Child Abuse: Preliminary Results From an Outpatient Therapy Program
Source: Front Psychiatry. 2020 Mar 3;11:88. doi: 10.3389/fpsyt.2020.00088 (PMC7063028; doi:10.3389/fpsyt.2020.00088)
Supplement: Supplementary file 3 [file Table_3.docx]

Table 3

Number of and reasons for drop-outs and expulsions during different phases of treatment

| Point in time | Number of and reasons for voluntary drop-outs  *N* = 49 | Number of and reasons for treatment expulsions  *N* = 10 |
| --- | --- | --- |
| During diagnostic phase | - comorbid disorder requiring treatment (*n =* 3)  - no motivation of change (*n* = 3)  - lack of problem awareness (*n* = 3)  - move to a new city (n = 1)  - economic reasons (n = 1)  - detention start (*n* = 1)  - n/A (*n* = 1 ) | - comorbid disorder requiring treatment (*n* = 1) |
| Before treatment start | - no motivation of change (*n* = 2)  - no psychological strain (*n* = 1) | - n/A (*n* = 1) |
| During group therapy | - no motivation of change (*n* = 4)  - economic reasons (*n* = 3)  - comorbid disorder requiring treatment (*n* = 3)  - lack of problem awareness (*n* = 2)  - move to a new city (*n* = 1)  - detention start (*n* = 1)  - no psychological strain (*n* = 1) | - comorbid disorder requiring treatment (*n* = 3)  - no motivation of change (*n* = 2) |
| During single therapy | - move to a new city (*n* = 3)  - n/A (*n = 3*)  - no motivation of change (*n* = 2)  -detention start (*n* = 2)  - economic reasons (*n* = 1)  - comorbid disorder requiring treatment (*n* = 1)  - no psychological strain (*n* = 1)  - lack of problem awareness (*n* = 1) | - no motivation of change (*n* = 2) |
| During single and group therapy | - no motivation of change (*n* = 2) | - comorbid disorder requiring treatment (*n* = 1) |
| After treatment/before post-assessment | n/A *(n* = 2) | - |

*Note*. Reasons for drop-outs and expulsions were based on participants’ self-reports or the therapists’ subjective perception. Whenever more than one reason was applicable, the reason considered most important was recorded. Economic reasons refer to scheduling problems or not being able to raise enough money for travel expenses. n/A = not available.
